# Ultrasonographic Classification of Achilles Tendon Ruptures as a Rationale for Individual Treatment Selection

**DOI:** 10.5402/2011/869703

**Published:** 2011-10-24

**Authors:** Michael H. Amlang, Hans Zwipp, Adina Friedrich, Adam Peaden, Alfred Bunk, Stefan Rammelt

**Affiliations:** ^1^Department of Trauma and Reconstructive Surgery, University Hospital Carl Gustav Carus, Fetscherstra*β*e 74, 01309 Dresden, Germany; ^2^Podiatric Medicine and Surgery Residency, Florida Hospital, East Orlando, Orlando, FL, USA; ^3^Department of General Surgery, University Hospital Carl Gustav Carus, Fetscherstra*β*e 74, 01309 Dresden, Germany

## Abstract

*Purpose*. This work introduces a distinct sonographic classification of Achilles tendon ruptures which has proven itself to be a reliable instrument for an individualized and differentiated therapy selection for patients who have suffered an Achilles tendon rupture. *Materials and Methods*. From January 1, 2000 to December 31, 2005, 273 patients who suffered from a complete subcutaneous rupture of the Achilles tendon (ASR) were clinically and sonographically evaluated. The sonographic classification was organized according to the location of the rupture, the contact of the tendon ends, and the structure of the interposition between the tendon ends. *Results*. In 266 of 273 (97.4%) patients the sonographic classification of the rupture of the Achilles tendon was recorded. Type 1 was detected in 54 patients (19.8%), type 2a in 68 (24.9%), type 2b in 33 (12.1%), type 3a in 20 (7.3%), type 3b in 61 (22.3%), type 4 in 20 (7.3%), and type 5 in 10 (3.7%). Of the patients with type 1 and fresh ASR, 96% (*n* = 47) were treated nonoperative-functionally, and 4% (*n* = 2) were treated by percutaneous suture with the Dresden instrument (pDI suture). Of the patients classified as type 2a with fresh ASR, 31 patients (48%) were treated nonoperatively-functionally and 33 patients (52%) with percutaneous suture with the Dresden instrument (pDI suture). Of the patients with type 3b and fresh ASR, 94% (*n* = 34) were treated by pDI suture and 6% (*n* = 2) by open suture according to Kirchmayr and Kessler. *Conclusion*. Unlike the clinical classification of the Achilles tendon rupture, the sonographic classification is a guide for deriving a graded and differentiated therapy from a broad spectrum of treatments.

## 1. Introduction

Twenty years ago, ultrasound was introduced as an image-providing procedure for the diagnostic evaluation of ruptures of the Achilles tendon. Today it is an established standard diagnostic instrument for the Achilles tendon [1–5]. The interruption of continuity can be located by the dynamic investigation of an abrogated power transmission between the triceps surae muscle and the calcaneal Achilles tendon insertion. By doing so, the area of the rupture can be located. 

Thermann et al. [6] introduced the sonographic evaluation of the behavior of the tendon ends at plantar flexion as a method for identifying the indication for nonoperative-functional therapy of the ruptured Achilles tendon. Nonoperative-functional therapy with Vario Stabil shoe was recommended if the distance of the tendon ends was maximally 5 mm in 20° plantar flexion as opposed to the physiological position [6, 7]. However, it appeared that due to the variable morphology of the rupture location a definition of the exact measuring points was possible but became very difficult to obtain reliable data. The experiences with the sonographic evaluation of the healing process after nonoperative-functional therapy, independent reexaminations, and the development of a minimally invasive suture technique were reasons for the development of an ultrasonic classification of the rupture of Achilles tendon, leading to a guideline for using a nonoperative-functional procedure [8, 9].

Using a distinct sonographic classification of the Achilles tendon rupture, the aim of this investigation was to evaluate the frequency of different rupture locations and contact types of the tendon ends in 20° plantar flexion.

## 2. Methods

### 2.1. Patients

From January 1, 2000 till December 31, 2005, 273 patients were clinically and sonographically diagnosed with a complete subcutaneous rupture of the Achilles tendon (ASR). 226 of these patients had a fresh rupture of the Achilles tendon (time between rupture and beginning of therapy within 72 hours), and 47 patients had an outdated or spontaneous rupture of Achilles tendon (Table 2). The sex distribution amounted to 233 men (85.3%) and 40 women (14.7%). The mean age of the patients with fresh rupture of the Achilles tendon was 44.7 (16–79) years. The mean age of patients with outdated or pathological rupture was 59.6 (29–90) years. The patients were supervised in a special consulting period and registered prospectively.

### 2.2. Method

**Figure 1:**
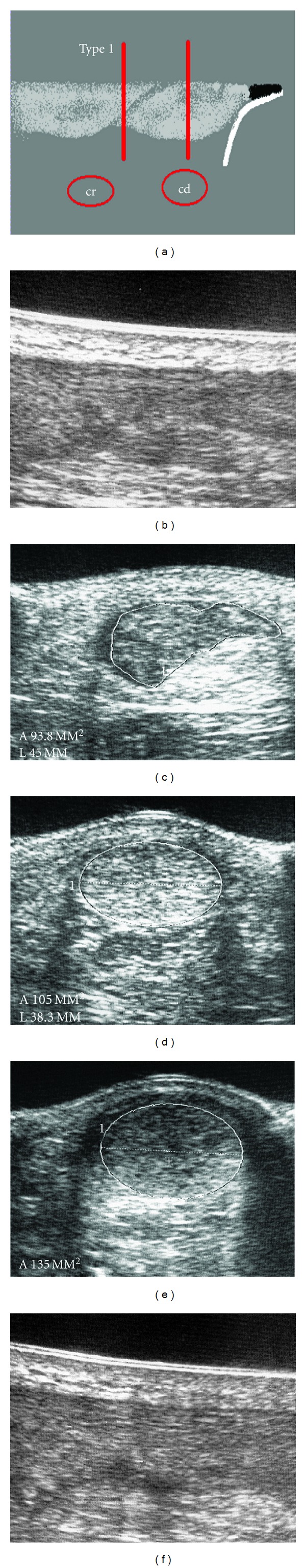
Type 1 rupture with high contact of the tendon ends. (a) Scheme. (b) Complete adaption in 20° plantar flexion in the longitudinal section. (c) Minimum cross-section area in the rupture zone, cr = 94 mm^2^. (d) Maximum cross-section area distal to the rupture, cd = 105 mm^2^, adaption value av^son^ = 90%. (e) Healed tendon after 8 weeks with nonoperative-functional therapy with a cross-section of 135 mm^2^. (f) Longitudinal section after 8 weeks.

**Figure 2:**
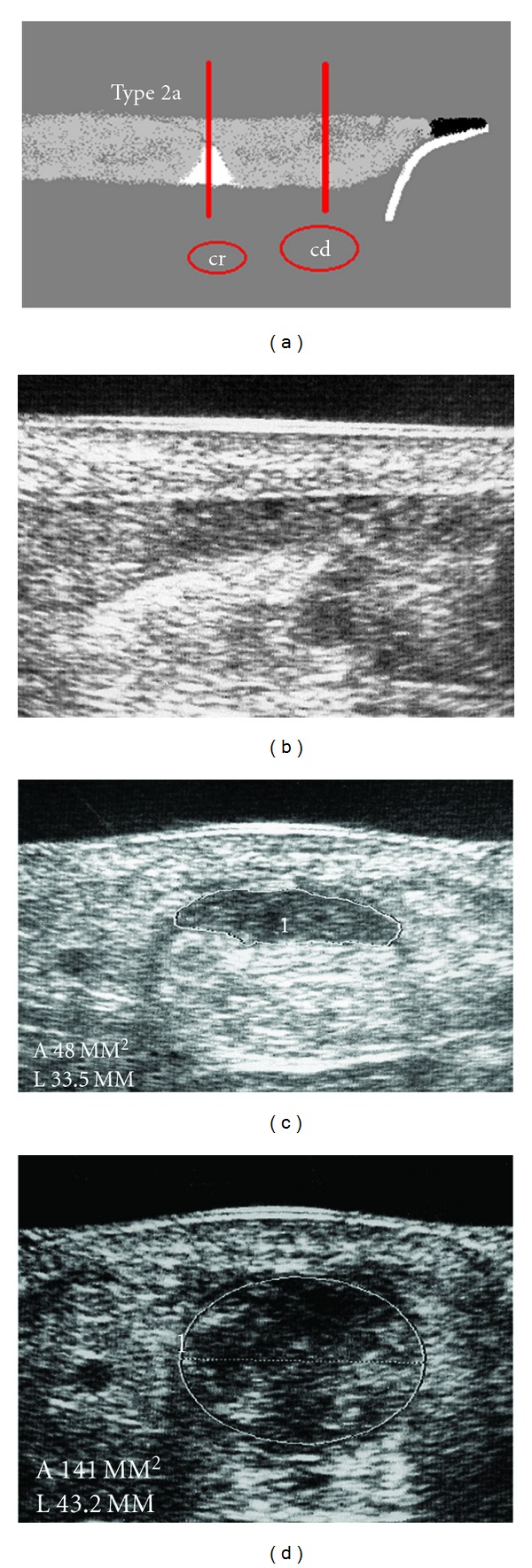
Type 2a rupture with partial contact of the tendon ends and interposed hyperechoic structure. (a) scheme. (b) Longitudinal section. (c) Minimum cross-section area in the rupture zone, cr = 48 mm^2^. (d) Maximum cross-section area distal to the rupture, cd = 141 mm^2^, adaption value av^son^ = 34%.

**Figure 3:**
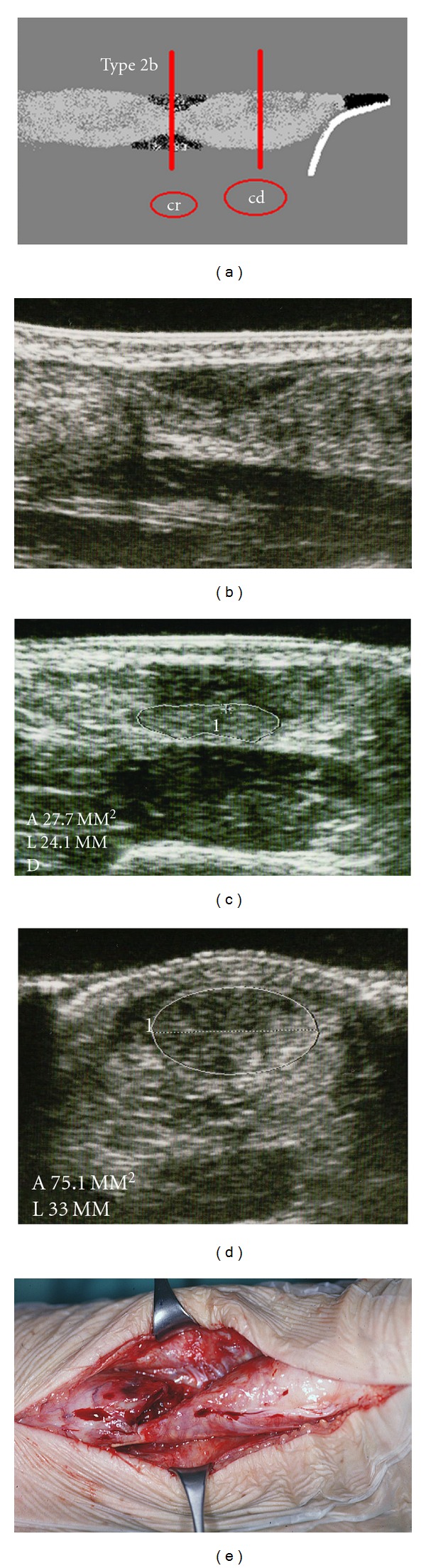
Type 2b rupture with partial contact of the tendon ends without interposed hyperechoic structure. (a) Scheme. (b) Longitudinal section. (c) Minimum cross-section area, cr = 28 mm^2^. (d) Maximum cross-section area distal to the rupture, cd = 75 mm^2^, adaption value av^son^ = 37%. (e) Clinical example of a “sand clock phenomenon” after nonoperative treatment with rerupture.

**Figure 4:**
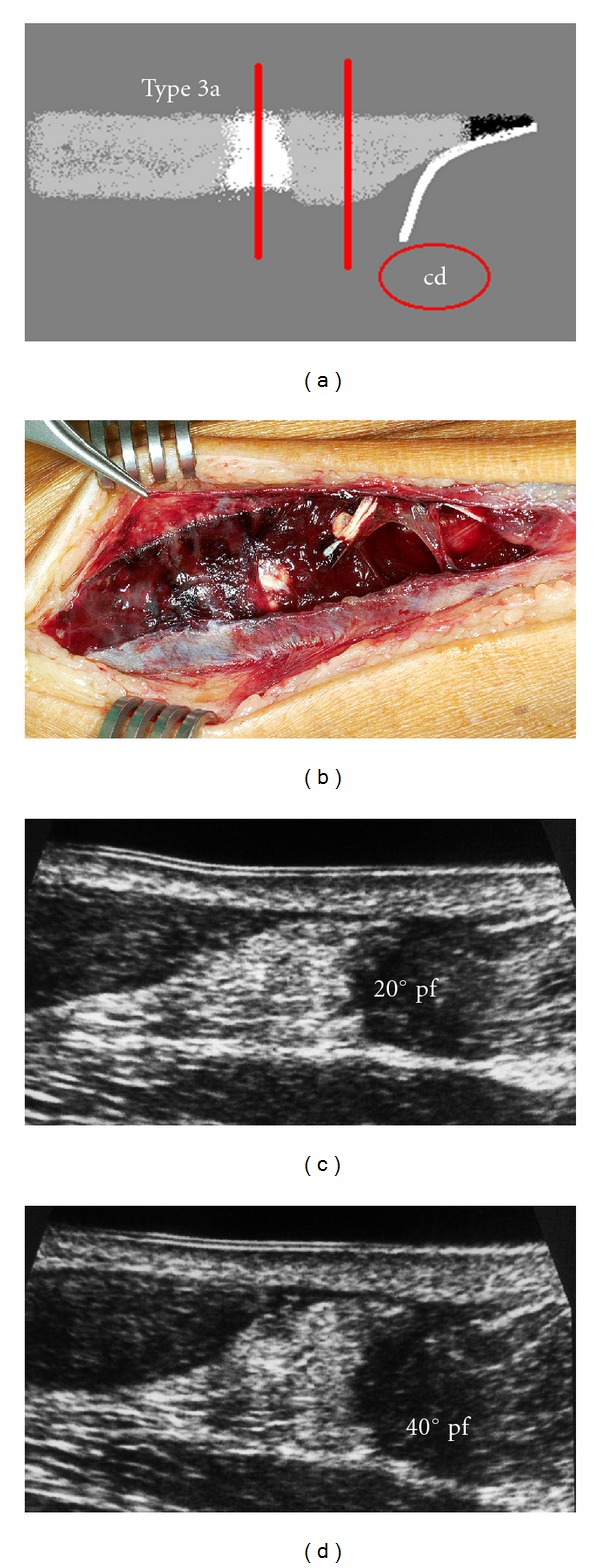
Type 3a rupture with dehiscene of the tendon ends and interposed hyperechoic structure. (a) Scheme. (b) Intraoperative example of a fresh type 3a rupture. (c) Longitudinal section with 20° plantar flexion. (d) Hematoma inhibits the stump adaption also in 40° plantar flexion.

**Figure 5:**
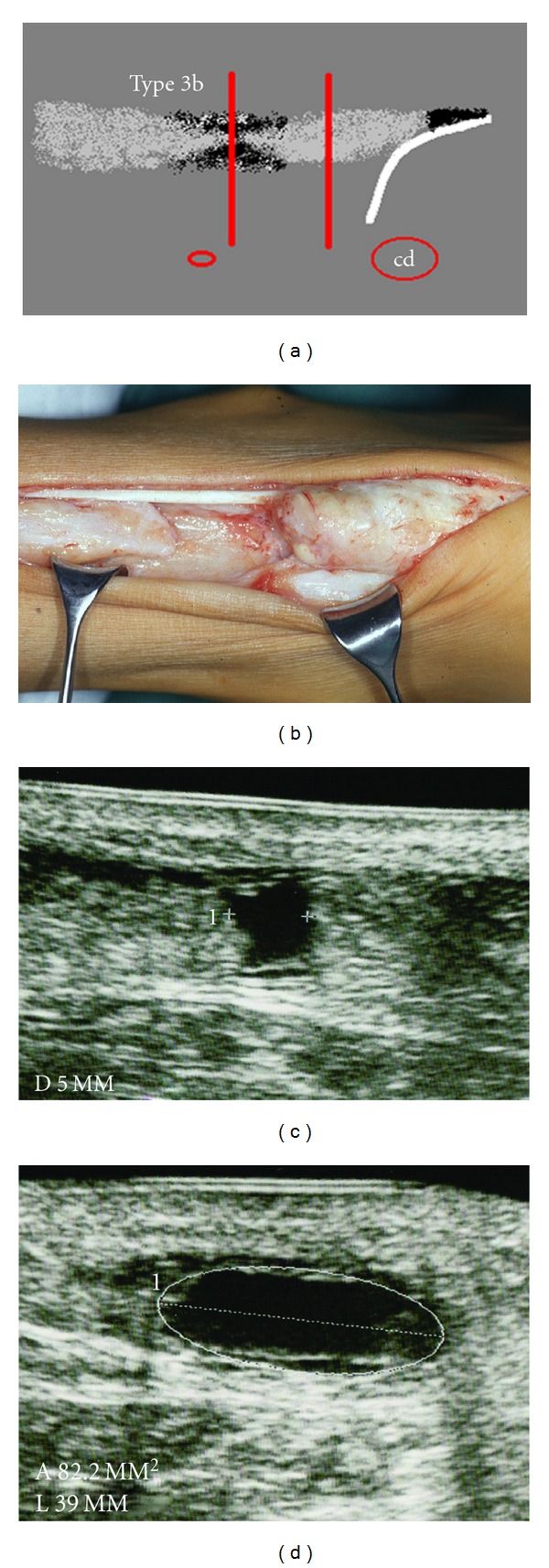
Type 3a rupture with a dehiscene of the tendon ends without interposed hyperechoic structure. (a) Scheme. (b) Intraoperative example with a chronic rupture. (c) Longitudinal section with an echo-free gap between the tendon ends. (d) Cross-section.

The sonographic classification took into consideration the location of the rupture, the contact of the tendon ends in 20° plantar flexion, and the structures between the tendon ends (Table 1, Figures (1)–(5)). The ultrasonic investigation was administered to the patients in prone position with 20° plantar flexion in the ankle joint. Equipment of the Toshiba company (A SSA-250A) with high-definition transducer assembly was used for close-up range (annular array 7.5 MHz with integrated delay block, model UIAA250A).

In the first step, the proof of interrupted continuity of the Achilles tendon took place via dynamic investigation. By passively moving the foot at the ankle joint, the abrogated power transmission on the triceps surae muscle was sonographically verified. Subsequently, after an orienting longitudinal exposure of the Achilles tendon with localization of the rupture, the determination of the contact behavior of the tendon ends was performed. In case of both clinically and sonographically clear findings, the analysis was performed by an examiner with little experience with the explained measuring method. A qualitative allocation to each category was then done. This included both complete and partial contacts as well as dehiscence.

The quantitative evaluation of the contact of the tendon ends was done by measuring the minimum cross-section area of the tendon ends in the rupture zone (cr) and via measurement of the maximum cross-section area distal to the rupture (cd) at 20° plantar flexion. The allocation was carried out quantitatively by the adapted value av^son^ = cr/cd∗100 (in %), that is, the relationship between the minimum cross-section area in the ruptured area to the maximum cross-section area distal to the rupture. Due to the physiological isthmic characteristic of the Achilles tendon, an adaptation value of more than 70% was defined as high contact of the tendon ends (Figure 1). 

Dehiscence of the tendon ends was valued at less than 30% (Figures 4 and 5). Adapted values from 30 to 70% were defined as middle or partial contact of the tendon ends (Figures 2 and 3). Due to the special characteristics of tendon healing of distal ruptures (type 5) and ruptures of the intersection of muscle and tendon (type 4) these were registered separately. Additionally, the cross-section area of the tendon of the contralateral limb was also measured in the isthmic area.

The structure of the interposed material between the tendon ends was assessed regarding its echogenicity. Organized hematoma was interposed as a hyperechoic structure and could secondarily be reconstituted into tendon tissue due to extrinsic tendon healing. By the evaluation of the rupture location, contact behavior of the tendon ends, and structure of the interposed substance, the classification of the 5 adaptation types (Table 1) was possible. In conjunction with clinical aspects surrounding each patient such as pathomechanism, symptomatology, concomitant diseases, achillodynia, and beginning of therapy, the decision for the appropriate therapy (Table 2) was made utilizing this sonographic classification.

### 2.3. Statistics

Differences between independent data (age, sex, and rupture side of patients with fresh and outdated or pathological rupture) were compared by unpaired Student's *t*-test. Nominal data were analyzed with Pearson chi-square statistics (SPSS for Windows 13.0, SPSS Software GmbH, Germany). A *P* < 0,05 was considered to be statistically significant.

## 3. Results

The age of patients with fresh rupture of the Achilles tendon (44.7 years, mean = 12.05) differed significantly from the group of patients with outdated or pathological rupture (59.6 years, mean = 12.55). No significant difference in the sex and the frequency distribution of the side of the rupture could be determined.

**Figure 6:**
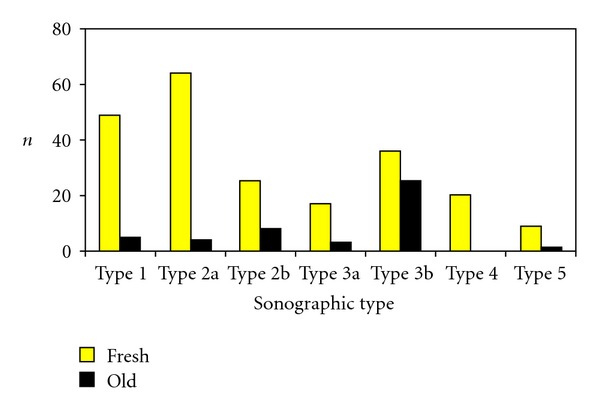
Frequency of the sonographic type in 266 patients with Achilles tendon rupture (fresh: fresh ruptures and old: outdated or pathological ruptures).

In 266 of 273 (97.4%) patients, the ultrasonic type (type of adaptation) of Achilles rupture was registered (Figure 5): Type 1 in 54 patients (19.8%), type 2a in 68 (24,9%), type 2b in 33 (12.1%), type 3a in 20 (7.3%), type 3b in 61 (22.3%), type 4 in 20 (7.3%), and type 5 in 10 (3.7%) (Figure 6). In 7 cases (2.6%) no allocation to an ultrasonic type could be made, which was usually due to an examiner not trusted with the method.

The ultrasonic type could be determined in 220 of 226 (97.3%) patients with fresh rupture of the Achilles tendon. Of these patients, 179 (81.4%) underwent the complete cross-sectional evaluation due to the initial sonographic appearance of the tendon. The classification of the other 41 (18.6%) patients occurred qualitatively also once the initial sonographic evaluation was complete.

In the case of 47 patients with outdated or pathological rupture of the Achilles tendon, an ultrasonic type could be determined in 46 (98%). The classification took place qualitatively in 27 (58%) patients. 19 patients (40%) were allocated quantitatively upon completion of the cross-sectional evaluation.

### 3.1. Fresh Rupture of Achilles Tendon (*n* = 220)

**Figure 7:**
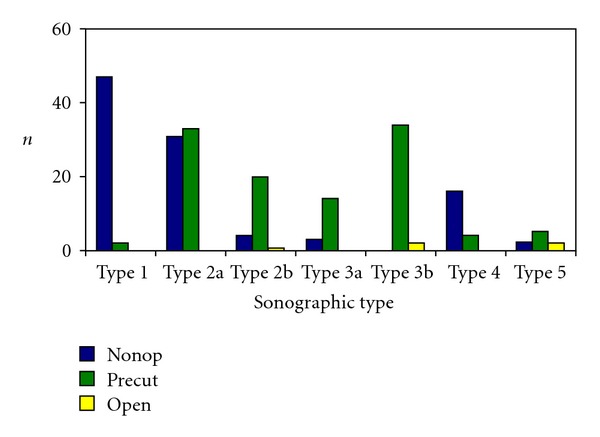
Ratio between sonographic type and therapy in 220 patients with fresh rupture (nonop: nonoperative, percut: percutaneous, and open: open repair).

Type 1 could be determined in 49 patients (22.3%) with fresh ASR. 47 patients (96%) were treated nonoperative-functionally with Vario Stabil shoe and 2 patients (4%) by percutaneous suture with the Dresden instrument (pDI suture) (Figure 7). Type 2a was determined in 64 patients (29.1%) with fresh ASR. Of these, 31 patients (48%) were treated nonoperative-functionally and 33 patients (52%) by percutaneous suture with the Dresden instrument (pDI seam). Type 2b was recorded in 25 patients (11.4%) with fresh ASR. Of these, 4 patients (16%) were treated nonoperative-functionally, 20 patients (80%) by pDI suture, and one patient (4%) with an open suture technique according to Kirchmayr and Kessler. Type 3a was observed in 17 patients (7.7%) with fresh ASR. Of these, 3 patients (18%) were treated nonoperative-functionally and 14 patients (82%) by pDI suture. Type 3b was registered in 36 patients (16.3%) with fresh ASR, 34 of which (94%) were treated by pDI suture and two (6%) with open suture according to Kirchmayr and Kessler.

Type 4 was determined in 20 patients (9.1%) with fresh ASR. Of these, 16 patients (80%) were treated nonoperative-functionally and 4 patients (20%) with pDI suture. Type 5 was registered in 9 patients (4.1%) with fresh ASR. Two of these patients (22%) were treated nonoperative-functionally, 5 patients (56%) with pDI suture, and two patients (22%) with open suture according to Kirchmayr and Kessler.

### 3.2. Outdated or Pathological Rupture of Achilles Tendon (*n* = 46)

**Figure 8:**
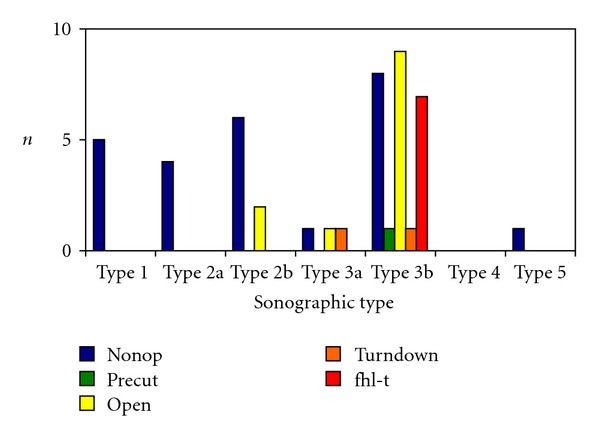
Ratio between sonographic type and therapy in 46 patients with outdated and pathological rupture (nonop: nonoperative, percut: percutaneous, open: open, turndown: turndown flap, and fhl-t: Flexor hallucis longus transfer).

Type 1 could be determined in 5 patients (11%) with outdated or pathological ASR. Four patients (80%) were treated nonoperative-functionally with Vario Stabil shoe and 1 patient (20%) functionally with a heel lift in the ready-made shoe (Figure 8).

Type 2a was registered in 4 patients (9%) with outdated or pathological ASR. All 4 patients (100%) were treated nonoperative-functionally. Type 2b was determined in 8 patients (17%) with outdated or pathological ASR. 6 patients (75%) were treated nonoperative-functionally and 2 patients (25%) with open suture according to Kirchmayr and Kessler. Type 3a was observed in 3 patients (6%) with outdated or pathological ASR. 1 patient (33%) was treated nonoperative-functionally, 1 patient (33%) with open suture, and 1 patient (33%) with a turndown technique according to Silfverskiöld. Type 3b was registered in 25 patients (54%) with outdated or pathological ASR. Of these, 6 patients (24%) were treated nonoperative-functionally, 2 patients (8%) only functionally, 1 patient (4%) by pDI suture, 9 (32%) with open suture according to Kirchmayr and Kessler, 1 (4%) with the turndown technique, and 7 (28%) with Flexor Hallucis Longus Tendon Transfer. Type 4 was not observed in patients with outdated or pathological ASR. Type 5 was noted in 1 patient (2%) with outdated or pathological ASR. This patient was treated nonoperative-functionally.

## 4. Discussion

The aim of therapy for ruptured Achilles tendons is healing of the tendon with complete functional restoration of the patient [12–14]. For this, multiple conditions are necessary, including avoidance of complications such as infection, re-rupture, or injury of the sural nerve and the formation of a homogeneous regenerated tendon with optimal preloading of the triceps surae muscle [9, 11–13]. In the literature, Mason and Allen have proven experimentally that only with sufficient contact of the tendon ends an unimpaired tendon healing can be expected [15].

The contact of the tendon ends can be clearly identified by means of sonography in 20° plantar flexion, the choice of the therapy can be considered, and the evaluation of the tendon healing in the treatment process can take place. The position of 20° plantar flexion of the foot corresponds to the position of the ankle joint, in which protection of the healing tendon in the first 6 weeks may occur [8, 13, 16]. If the contact of the tendon ends is inadequate from the start, it cannot be expected that under nonoperative therapy this will spontaneously improve. Besides, no improvement of the chord adaptation can be achieved by surgical treatment when, via sonographic evaluation, complete contact of the tendon ends is evident.

The spectrum of the presently used therapy methods for the rupture of the Achilles tendon is multifacetted [11, 12, 14]. With all forms of therapy, an optimal, functional result can be achieved under suitable conditions with an uncomplicated healing process [12]. Nevertheless, the risk profile of the different forms of therapy is very distinct. 

The high rate of re-ruptures under nonoperative-immobilizing therapy and the high requirements of patient compliance under nonoperative-functional therapy are the main problems in nonsurgical treatment [12]. An infection of the tendon after open suture technique is a complication which can be controlled with great difficulty. This frequently leads to the loss of the Achilles tendon and may require plastic surgery techniques for covering of the Achilles tendon region [10]. A current prospective, randomized study with 60 patients with open suture according to Krackow or turndown surgical treatment according to Silfverskiöld reports over 2 deep and 4 superficial infections of the wound as well as over 6 re-ruptures. This corresponds with a complication rate of 20% [14]. Lim et al. [17] showed in a prospective, randomized study a significantly smaller infection rate in patients who underwent percutaneous surgery with a small modified Ma-Griffith-technology in relation to the open suture according to Kessler with monofilament polydioxanone fiber of the strength 1. But not each patient is suitable for a minimally invasive suture technique, and nonoperative therapy cannot lead to proper healing of the tendon when the tendon ends are dehiscent (Figure 5).

The groups of patients with fresh, outdated, or pathological rupture must be clearly separated regarding the therapy decision (Table 2). This reflects both the significant disparity in age and the therapy modalities that are utilized. In the elder rupture of the Achilles tendon, a simple rearrangement of the contact of the tendon ends is not sufficient, since the tendon ends are no longer able to regenerate and a shortening of the triceps surae is present (Figure 5). In contrast, a fresh rupture of the Achilles tendon with good contact of the tendon ends either with nonoperative-functional therapy or after minimal invasive suture immigrating of fibroblasts can occur with resulting collagen formation, thereby establishing a homogeneous tendon in the context of extrinsic tendon healing [8, 16].

In 81.4% of the patients with fresh ASR, the quantification of the contact of the tendon ends took place via the determination of the minimum cross-section in the ruptured area and the maximum cross-section distal to the rupture. In obvious cases, a well-defined classification to the ultrasonic types could also take place on the basis of the ultrasonic longitudinal section. When distal ASR (type 4) and proximal ASR (type 5) exist, the qualitative allocation to the adaptation types by ultrasonic localization of the rupture is sufficient. Also when a clear dehiscence of the tendon ends is detected, a direct, ultrasonic allocation can also take place to the type 3b, whereby the description of the size of the defect in the longitudinal profile is important for the surgeon.

In type 1, which was identified in approximately one fifth of the cases, the tendon ends are well approximated without a gap. The definition of an adaptation value for type 1 of over 70% considers the physiological isthmic formation of the tendon (Figure 1). The indication for nonoperative-functional therapy is limited to compliant patients with complete contact of the tendon ends (type 1) only. Nonoperative-functional therapy requires high compliance, an experienced team of therapists consisting of a competent surgeon with experience in ultrasonic usage, a skillful orthopedic shoemaker, and physiotherapists who are specifically trained in this particular disease pattern. Upon patient request or in the case of noncompliance, the percutaneous suture with the Dresden instrument (pDI suture) was utilized in this classification of injury. The pDI suture is a minimal invasive, closed, subfascial surgical technique which preserves the peritendon and minimizes the risks of other minimally invasive techniques such as injury to the Sural nerve or worsening of the contact of the tendon ends by the suture [8].

Partial contact of the tendon ends (type 2) is the most frequent type of adaptation at 37% and can be easily observed sonographically. Usually a small, organized hematoma (type 2a) is located between the tendon ends and is shown as an hyperechoic structure. This hematoma reconstitutes into tendon tissue, which can be detected via sonography between the 6th and 8th weeks after rupture (Figure 2). Nonoperative-functional therapy can be used in this type; however, our procedure of the pDI suture worked satisfactorily and is recommended as an alternative since this does not require strict compliance.

Utilizing nonoperative-functional therapy in the Achilles tendon rupture with partial tendon contact but without differentiated, interposed substance (type 2b), a depleted zone in the area of rupture can occur after the healing process has completed. This is referred to as the so-called sand clock phenomenon (Figure 3). This area with a clearly decreased cross-sectional area is the most common point for the occurrence of a re-rupture and an area of suspicion for relevant elongation of the tendon.

If a dehiscence of the tendon ends (type 3) is detectable, which corresponds with the definition of an adaptation value less than 30%, surgical rearrangement of the tendon continuity should proceed (Figure 4). Serous or serosanguinous liquid in the area of rupture (type 3b) in the ultrasonic control investigation will reveal no increase of the echo density in the area of rupture in the future. These substances are not subject to a differentiation to tendon tissue, and the spontaneous rearrangement of tendon continuity fails to appear (Figure 5).

If an extended hematoma obstructs the contact of the tendon ends, under nonoperative therapy healing in elongation or a prolonged restructuring of the interposed material can be observed (Figure 4). In patients with a fresh rupture type 3a, the minimal invasive suture (pDI suture) was used in 82% and in type 3b in 94% of the cases (Figure 4). In an outdated rupture with dehiscence of the tendon, only an open reconstruction is useful due to the shortening of the triceps surae muscle and the induration of the tendon stumps [12, 18]. If a dehiscence of over 5 cm and a pronounced degeneration of the tendon tissue was noted, replacement of the Achilles tendon was used with Flexor Hallucis Longus Tendon Transfer (FHL transfer) [18].

Older patients with concomitant diseases such as diabetes mellitus, problematic skin conditions, and pathological ruptures, for example under immunosuppression, benefit even from the nonoperative-functional treatment when an insufficient contact of the tendon ends was registered, since a potential force reduction under nonoperative-functional treatment is to be regarded as the smaller disadvantage in relation to the high infection risk under surgical treatment. To what extent the minimally invasive suture technique becomes an auspicious alternative in these patients is still in clinical observation. The ability of the patient to use a force approximately 50% of the plantar flexion of the Achilles tendon is expected even if loss of the Achilles tendon has occurred [19].

The rupture at the intersection between muscle and tendon (type 4) possesses a high healing potency due to the quality of blood circulation based on the proximity to the musculature. In 80% of these cases, nonoperative-functional therapy was utilized. Alternatively the minimally invasive suture (pDI suture) was implemented in 20% of these patients since fewer requirements regarding patient compliance are necessary. The positive healing behavior of proximal ruptures becomes obvious due to the fact that no cases of outdated rupture were found in this location.

Distal rupture of Achilles tendon (type 5) is a problematic location due to the regular incidence of insertional Achilles tendinosis and the decreased blood supply to the area. Even with incidence of this tendinosis of the insertion in a fresh, distal rupture, good experiences were seen with the transcalcaneal pDI suture technique [8]. In patients with an elder type 5 rupture using our own procedure, the replacement of the degenerated, distal Achilles tendon by a Flexor Hallucis Longus transfer worked satisfactorily with the aim of a functional reestablishment of the patient's plantarflexory abilities [18].

It is evident that with real-time sonography by dynamic investigation, the differentiation between a partial and a complete rupture of the Achilles tendon can be performed reliably. “Partial rupture of the Achilles tendon” should not confused with a near total rupture of the tendon, but it refers more to the elongated and mechanically insufficient remaining fibers. In a typical presentation, the diagnosis of “partial rupture of the Achilles tendon” is often an incorrect one because the unrestricted mobility of the foot and assumed remaining continuity in the MRI of the intact Plantaris muscle tendon are regarded as proof for merely a partial rupture. In partial rupture of the Achilles tendon after a suddenly arising pain in the area, no defect can be recognized by the clinical investigation, the Thompson test is negative, and the ultrasound reveals a normal power transmission of the triceps surae muscle during passive dorsiflexion of the foot.

Ultrasound of the Achilles tendon is further indicated for repeat evaluation of tendon healing and the differential diagnoses of the so-called achillodynia [2, 5, 13, 20–26].

## 5. Conclusion

In conclusion it can be stated that ultrasound has become essential both in the diagnosis of the rupture of the Achilles tendon and in the selection of therapy for the injury. The sonographic classification is, in addition to being a means for the clinical characterization of the rupture, a reliable tool for deriving an individualized, differentiated therapy pattern from a varied spectrum of methods of treatment.

## Figures and Tables

**Table 1:** Sonographic classification of the Achilles tendon ruptures according to the location, the contact of the tendon ends in 20° plantar flexion, and the structure between the tendon ends (cr: minimum cross-section area of the tendon ends in the rupture zone; cd: maximum cross-section area distal to the rupture; n.m.: not measured).

	Sonographic type	Adaptation value (av^son^)	Characteristics
1	High contact	cr/cd∗100 > 70%	Complete adaption of the tendon ends in 20° plantar flexion

2a	Partial contact with hyperechoic interposed structure	cr/cd∗100 = 30–70%	Partial adaption with organized hematoma between the tendon ends

2b	Partial contact without hyperechoic interposed structure	cr/cd∗100 = 30–70%	Partial adaption without organized hematoma between the tendon ends

3a	Dehiscence with hyperechoic interposed structure	cr/cd∗100 < 30%	Organized hematoma between the tendon ends

3b	Dehiscence without hyperechoic interposed structure	cr/cd∗100 < 30%	Gap between the tendon ends

4	Proximal rupture	n.m.	Rupture at the intersection of muscle and tendon

5	Distal rupture	n.m.	Near insertion, often in cases with insertional tendinopathy

**Table 2:** Clinical classification of the Achilles tendon rupture (^1^splint or cast in plantar flexion).

Clinical type	Characteristics	*n* = 273
Acute (fresh) rupture [10]	(i) Typical history with rupture event and sudden pain (ii) Therapy^1^ starts within the first 72 hours after the rupture	226 (82,8%)
Pathological (spontaneous) rupture	(i) Untypical history without rupture event (ii) Low or no pain (iii) Calf-squeeze test showed complete rupture	25 (9,2%)

Outdated rupture		22 (8%)
* Subacute rupture [10]*	(i)* Typical history with rupture event and sudden pain * (ii)* Therapy^1^ between day 4 and week 4 after the rupture event *	*5 (1,8%)*
* Chronic rupture [11]*	(i)* Typical history with rupture event and sudden pain * (ii)* Therapy 4 weeks and more after the rupture event *	*17 (6,2%)*
